# Oestradiol Contributes to Differential Antitumour Effects of Adjuvant Zoledronic Acid Observed Between Pre- and Post-Menopausal Women

**DOI:** 10.3389/fendo.2021.749428

**Published:** 2021-10-18

**Authors:** Victor G. Canuas-Landero, Christopher N. George, Diane V. Lefley, Hannah Corness, Munitta Muthana, Caroline Wilson, Penelope D. Ottewell

**Affiliations:** Department of Oncology and Metabolism, The Mellanby Centre for Musculoskeletal Research, The University of Sheffield, Sheffield, United Kingdom

**Keywords:** breast cancer, zoledronic acid, oestradiol, menopause, bone microenvironment

## Abstract

Clinical trials have demonstrated that adding zoledronic acid (Zol) to (neo)adjuvant standard of care has differential antitumour effects in pre- and post-menopausal women: Both benefit from reduced recurrence in bone; however, while postmenopausal women also incur survival benefit, none is seen in premenopausal women treated with adjuvant bisphosphonates. In the current study, we have used mouse models to investigate the role of oestradiol in modulating potential antitumour effects of Zol. Pre-, peri-, and post-menopausal concentrations of oestradiol were modelled in BALB/c wild-type, BALB/c nude, and C57BL/6 mice by ovariectomy followed by supplementation with oestradiol. Mice also received 40 mg/kg/day goserelin to prevent ovariectomy-induced increases in follicle-stimulating hormone (FSH). Metastasis was modelled following injection of MDA-MB-231, 4T1, or E0771 cells after ovariectomy and saline or 100 μg/kg Zol administered weekly. Supplementing ovariectomised mice with 12.5 mg/ml, 1.38 mg/ml, and 0 ng/ml oestradiol, in the presence of goserelin, resulted in serum concentrations of 153.16 ± 18.10 pg/ml, 48.64 ± 18.44 pg/ml, and 1.00 ± 0.27 pg/ml oestradiol, which are equivalent to concentrations found in pre-, peri-, and post-menopausal humans. Osteoclast activity was increased 1.5–1.8-fold with peri- and post-menopausal compared with premenopausal oestradiol, resulting in a 1.34–1.69-fold reduction in trabecular bone. Zol increased trabecular bone in all groups but did not restore bone to volumes observed under premenopausal conditions. In tumour-bearing mice, Zol reduced bone metastases in BALB/c (wild-type and nude), with greatest effects seen under pre- and post-menopausal concentrations of oestradiol. Zol did not affect soft tissue metastases in immunocompetent BALB/c mice but increased metastases 3.95-fold in C57BL/6 mice under premenopausal concentrations of oestradiol. In contrast, Zol significantly reduced soft tissue metastases 2.07 and 4.69-fold in immunocompetent BALB/c and C57BL/6 mice under postmenopausal oestradiol, mirroring the results of the clinical trials of (neo)adjuvant bisphosphonates. No effects on soft tissue metastases were observed in immunocompromised mice, and differences in antitumour response did not correlate with *musculoaponeurotic fibrosarcoma (MAF)*, *macrophage capping protein (CAPG)*, or *PDZ domain containing protein GIPC1 (GIPC1)* expression. In conclusion, oestradiol contributes to altered antitumour effects of Zol observed between pre- and post-menopausal women. However, other immunological/microenvironmental factors are also likely to contribute to this phenomenon.

## Introduction

Bone is the predominant site for breast cancer metastases (1), and relapse most frequently occurs decades after the primary tumour has been removed. It is therefore presumed that disseminated tumour cells (DTCs) stay in a dormant state, possibly within the bone, for many years (2). The bone marrow microenvironment can influence these tumour cells to remain dormant, reactivate to form overt metastases or to be shed back into the bloodstream from which they can seed other organs (2, 3). Importantly, the bone microenvironment can be altered by both the bone-targeting bisphosphonates (4–6) and reproductive hormones such as oestradiol (7–9). Bisphosphonates, such as zoledronic acid (Zol), can affect cells within the bone microenvironment and influence the ability of tumour cells to establish in bone as metastases. In clinical studies, Zol decreased the number of DTCs in bone marrow aspirates from breast cancer patients (10–12), suggesting the DTCs had been killed, moved to another site in the body, or had entered a state of dormancy. Oestradiol has differential effects on the bone environment to Zol, actively promoting osteoblast-mediated bone formation (13). This, in addition to multiple additional effects on the vasculature, immune cells, bone marrow progenitors, haematopoetic stem cells, and cancer-associated fibroblasts [reviewed in (14, 15)], may be a mechanism by which oestradiol inhibits antitumour effects of Zol in premenopausal patients.

The preclinical and clinical evaluation of the antitumour effects of Zol commenced over a decade ago, but the first suggestion of interplay between Zol, menopausal status, and breast cancer recurrence only emerged in 2011 when reported by the ABCSG-12 and AZURE studies (16, 17). The AZURE study, which randomized 3,360 patients to receive 4 mg Zol for 5 years, showed a reduction in breast cancer recurrence with Zol in women who were >5 years postmenopausal (17). More recently, the 10-year follow-up data have shown a persistent increase in disease-free survival events outside of bone in women <40 years of age receiving Zol (18), suggesting that, in younger women with high levels of reproductive hormones, Zol may displace tumour cells out of the bone to distant sites. A meta-analysis of individual patient data from 18,766 women treated in breast cancer clinical trials with adjuvant bisphosphonates confirmed that postmenopausal women (defined clinically) had reduced recurrence rates at all distant sites (RR 0.82, 0.74–0.92; 2p = 0.0003), in bone specifically (0·72, 0·60–0·86; 2p = 0·0002), and improved overall breast cancer mortality (0.82, 0.73–0.93; 2p = 0.002) (19), but the increase in distant non-bone recurrence in premenopausal women treated with bisphosphonates was not demonstrated as it has been in the AZURE trial.

There is emerging evidence that oestradiol is a key hormone that can influence sites of breast cancer metastases. As part of the AZURE trial, a large number of baseline serum samples were collected and evaluated for baseline hormone levels [oestradiol, follicle-stimulating hormone (FSH), and inhibin]. These data showed an association between oestradiol levels of <50 pmol/l (postmenopausal range) and a significantly shorter invasive disease-free survival compared to an oestradiol level of ≥50 pmol/l [hazard ratio (HR) = 1.36; 95% CI 1.05–1.78; p = 0.022] (20). This was driven by more distant recurrences (outside of bone), regardless whether the primacy tumour was oestradiol receptor-positive (ER+ve) or -negative (ER-ve), indicating that very low levels of oestradiol may make the bone less attractive to DTCs, and therefore, they seek sites outside of bone in which to establish as metastatic disease. In support of this, *in vivo* studies have shown that supplementing mice with a 17β-oestradiol pellet enhanced spontaneous metastasis of ER+ve cells to bone (13), suggesting that high oestradiol may attract tumour cells to bone and may influence whether they survive/become dormant cells in the bone microenvironment or disseminate to other organs.

The current study aimed to identify the mechanisms by which oestradiol and Zol may interact to affect the bone microenvironment and tumour growth in bone and non-bone sites. To achieve this, we modelled physiologically relevant levels of oestradiol: 1) very low levels seen in aromatase inhibitor use in postmenopausal women; 2) low levels that we would expect in perimenopausal women; and 3) higher levels that we would find in premenopausal women in mice in the absence of tumour cells or before tumour cell dissemination into the circulation ± Zol.

## Materials and Methods

### Cell Culture

Cell lines used were as follows: human triple-negative MDA-MB-231 breast cancer cells (European Collection of Authenticated Cell Cultures, Wiltshire, UK) transfected with green fluorescent protein (GFP) and Luc2 (21) and triple-negative mouse mammary 4T1 and E0771 cells (kindly donated by Dr. Amy Kwan, University of Sheffield, Sheffield, UK, and Dr. Sandra McAllister, Harvard Medical School, USA, respectively) transfected with Luc2 (22, 23). MDA-MB-231 and 4T1 cell lines were maintained in Dulbecco’s modified Eagle’s medium (DMEM, Gibco, UK) supplemented with 10% fetal bovine serum (FBS; Gibco, UK), whereas E0771 cells were maintained in RPMI (Gibco, UK) supplemented with 10% FBS. MDA-MB-231-GFP-Luc2 was used within 30 passages, and 4T1-Luc2 and E0771-Luc2 were used within 20 passages after receipt.

### Generating Bone Metastatic 4T1 Cells

Here, 5 × 10^4^ 4T1-Luc2 cells were grown in DMEM + 10% FBS, washed, and prepared for intracardiac (i.c.) injection into 12-week-old female BALB/c mice (n = 5) (Charles River, UK). Tumour growth was monitored by an IVIS Lumina II system (Calliper Life Sciences, UK). Animals were sacrificed 2 weeks after injection of tumour cells. All tumour-bearing bones were isolated in a sterile environment and dissociated aseptically to collect 4T1-luc cells using Miltenyi tumour cell dissociation kit (Miltenyi Biotech, Germany). Selection of 4T1-luc cells was carried out using 60 μmol/L 6-thioguanine. Cultured colonies were grown and used for further *in vivo* experiments.

### *In Vivo* Studies

Experiments used 12-week-old female BALB/c wild-type, BALB/c ^fox/-^ nude, and C57BL/6 mice (Charles River, Kent, UK) kept on a 12-h/12-h light/dark cycle with free access to food and water. All studies were carried out in accordance with local guidelines and with Home Office approval under project licences 70/8964 and P99922A2E, University of Sheffield, UK.

Modelling of human pre-, peri-, and post-menopausal concentrations of oestradiol only was established by ovariectomising (OVX) mice to negate effects of other ovarian hormones, i.e., inhibin [as previously described (24, 25)] prior to supplementation with 17β-oestradiol (Sigma Aldrich, Poole, UK) at 12.5, 1.38, or 0 mg/L *via* their drinking water. To prevent predicted release of oestradiol from other tissues including adipose tissue as well as increases in FSH, 40 µg/kg/day goserelin (as goserelin acetate, Sigma Aldrich, UK) was administered *via* subcutaneous injection. Success of OVX ± oestradiol supplementation was assessed by imaging and weighing of mouse uterus at the end of each procedure (**Supplementary Figure S1**) and checking serum concentrations of oestradiol and FSH by ELISA. For experiments designed to investigate the effects of Zol on the bone microenvironment in the absence of tumours, 100 µg/kg Zol (supplied as disodium salt by Novartis) or phosphate buffered saline (PBS; control) was administered once per week starting 7 days after OVX (n = 5/group). Mice were sacrificed 14 days after OVX, and serum was collected and stored at -80°C for downstream analysis by ELISA, tibiae and femora were fixed in 4% paraformaldehyde (PFA; Fisher Scientific, UK) for μCT analysis before decalcification in 0.5 M EDTA and processing for histology (**Supplementary Figure S2A**).

To investigate the effects of Zol on tumour distribution under established pre-, post-, and peri-menopausal concentrations of oestradiol, 1 × 10^5^ MDA-MB-231-GFP-Luc2 cells were injected into the left cardiac ventricle (i.c.) of BALB/c^fox/-^ nude mice 3 days after OVX ± oestradiol + goserelin; or 5 × 10^4^ 4T1-Luc2 cells were injected i.c. into BALB/c mice 6 days following OVX ± oestradiol + goserelin; or C57BL/6 mice were injected with 1 × 10^5^ E0771-Luc2 i.c. 7 days following OVX ± oestradiol + goserelin. At 3 days after injection of tumour cells, 100 µg/kg/week Zol or PBS (control) was injected subcutaneously (s.c.) to the corresponding groups (n = 10/group) until the end of the experiment. BALB/c ^fox/-^ nude, BALB/c, and C57BL/6 mice were sacrificed 28, 15, and 19 days after OVX, respectively. Serum was collected and stored at -80°C for downstream analysis by ELISA, tibiae and femora were fixed in 4% PFA for μCT analysis before decalcification in 0.5 M EDTA and processing for histology (**Supplementary Figures S2B, C**).

### *In Vivo* Tumour Measurement

Tumour growth was monitored, in live mice, using an IVIS Lumina II system (Caliper Life Sciences, UK). Here, 30 mg/kg of D-Luciferin (Invitrogen, UK) was injected s.c. 4 min before imaging. Mice were imaged by dorsal and ventral exposure. Hind limbs and internal organs were extracted for additional *ex vivo* imaging after termination of the experiment. All images were analysed by creating a region of interest (ROI) around the tumour(s); luminescence signal was acquired in radiance (photons/second).

### Microcomputed Tomography Imaging

Microcomputed tomography (μCT) analysis was carried out on fixed tibiae using a Skyscan 1172 x-ray–computed microtomography scanner (Skyscan, Aartselaar, Belgium) equipped with an x-ray tube (voltage, 49 kV; current, 200 μA) and a 0.5-mm aluminium filter. Pixel size was set to 4.3 μm, and scanning initiated from the top of the proximal tibia as previously descried (26).

### Bone Histology

Tibiae and femora were fixed in 4% PFA for a minimum of 48 h prior to decalcification with 0.5 M EDTA at pH 7.0 for 3 weeks, changing the solution at weekly intervals. Bones were embedded in paraffin wax, and sections of 3 µm were taken using a microtome. Osteoclast identification was carried out by tartrate-resistant acid phosphatase (TRAcP) for consistency staining. Osteoblasts were identified as mononuclear cuboidal cells residing in chains along the bone surface. The number of osteoclasts and osteoblasts was analysed per millimetre in the cortical–endosteal bone and trabecular bone surfaces. Leica RMRB upright microscope and OsteoMeasure software (Osteometrics Inc.) were used for osteoclast and osteoblast scoring (27).

### Biochemical Analysis

Serum concentrations of Tartrate-Resistant Acid Phosphotase 5b (TRAcP 5b), procollagen 1 intact N-terminal propeptide (P1NP), oestradiol, and FSH were measured using commercially available ELISA kits: MouseTRAP™ Assay (Immunodiagnostic Systems), Rat/Mouse P1NP competitive immunoassay kit (Immunodiagnostic Systems), Mouse/Rat oestradiol ELISA (CALBIOTECH), and Mouse FSH (Elabsciences) and standard protocols, respectively.

### Gene Expression

Total RNA was extracted using an RNeasy kit (Qiagen) and manufacturers’ instructions. For Affymetrix array analysis, RNA was amplified using LunaScript RT SuperMix Kit (New England BioLabs, USA) before being reverse transcribed into cDNA using Superscript III (Invitrogen AB). Gene expression was assessed by analysing the number of cycles (CT) taken for expression of *glyceraldehyde 3-phosphate dehydrogenase* (*GAPDH*; control) *musculoaponeurotic fibrosarcoma* (*MAF*), *macrophage capping protein* (*CAPG*), and *PDZ domain containing protein GIPC1* (*GIPC1*) to be detected using the following assays: Hs00894322 for *GAPDH*, Hs01533575 for *MAF*, Hs 05867157 for *CAPG*, and Hs Hs01149347 for GIPC1 in human MDA-MB-231 cells and Mm00186822 for *GAPDH*, Mm0300214 for *MAF*, Mm00179028 for *CAPG*, and Mm00252127 for mouse 4T1 and E0771. All PCR assays were performed using an ABI 7900 PCR System (Perkin Elmer, Foster City, CA) and Taqman universal master mix (all reagents were purchased from Applied Biosystems *via* Thermofisher, UK).

### Statistical Analysis

Statistical analysis was performed using Prism GraphPad (version 8.0). Data are represented as mean ± SEM. Statistical significance was measured using parametric testing, assuming equal variance. Analysis was by one-way analysis of variance (ANOVA) followed by Newman–Keuls multiple comparison test, and standard unpaired t-tests were used to assess the difference between test and control samples. Statistical significance was defined as p ≤ 0.05. All graphs represent mean ± SEM, *p < 0.05, **p < 0.01, ***p < 0.001, ****p < 0.0001.

## Results

### Effects of Oestradiol and Zoledronic Acid on the Bone Microenvironment

**Figure 1:**
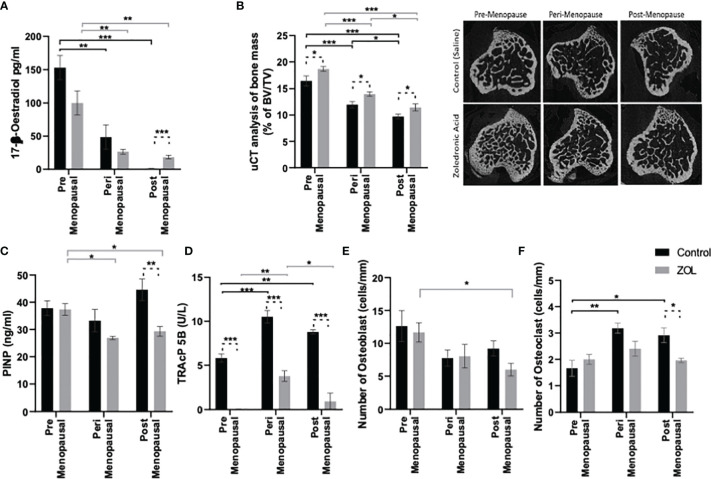
Modelling pre-, post-, and peri-menopausal concentrations of oestrogen ± zoledronic acid in mice. Twelve-week-old immunocompetent BALB/c mice underwent ovariectomy, and 0, 1.38, or 12.5 mg/L oestradiol was immediately supplemented *via* their drinking water. Three days later, five mice from each group received subcutaneous injection of 100 μg/kg zoledronic acid or saline control. All mice received a daily injection of 40 μg/kg goserelin. All data shown are 2 weeks following ovariectomy/oestradiol supplementation. **(A)** shows circulating concentrations of oestradiol in the plasma, as measured by ELISA. **(B)** shows the percentage of trabecular bone volume compared with tissue volume (TV/BV%) and radiomicrographs from representative μCT images. **(C, D)** shows effects on osteoblast and osteoclast activity, as measured by procollagen 1 intact N-terminal propeptide (P1NP) and tartrate-resistant acid phosphatase (TRAP) ELISA, respectively. **(E, F)** show effects on numbers of osteoclasts and osteoblasts that line trabecular and cortical bone per mm, respectively. All data on histograms are represented as mean ± SD; *p < 0.05, **p < 0.01, and ***p < 0.001.

Our hypothesis is that oestradiol-induced changes to the bone microenvironment affect metastatic outgrowth in bone and soft tissue. As female mice have low circulating concentrations of oestradiol compared with humans [65.94 ± 25.28 pg/ml in 14-week-old mice (**Supplementary Figure S3**) compared with 40–400 in premenopausal women] and do not naturally undergo menopause, we used OVX to establish low baseline concentrations of oestradiol and inhibin. This procedure did not reduce oestradiol release from other endocrine organs; therefore, mice also received 40 μg/kg/day goserelin to further reduce circulating concentrations of oestradiol to 1.01 ± 0.27 pg/ml (**Supplementary Figure S3**) and prevent OVX-induced release of FSH (**Supplementary Figure S4**). Mice were subsequently supplemented with 17β-oestradiol in their drinking water to establish concentrations of this hormone that are representative of pre-, post-, and peri-menopausal concentrations observed in women. Supplementation with 12.5, 1.38, or 0 mg/L resulted in serum concentrations of 153.16 ± 18.10, 48.64 ± 18.44, and 1.00 ± 0.27 pg/ml, respectively (**Figure 1A**). These concentrations are comparable to the 40–400, 22–120, and >30 pg/ml found in pre-, peri-, and post-menopausal women. Oestradiol concentrations were not significantly altered following administration of Zol (**Figure 1A**), and serum FSH concentrations remained below the level of detection by ELISA in all mice (**Supplementary Figure S4**). Removing oestrogen by OVX and administration of goserelin caused withering of mouse uterus and a significant reduction in uterus weight (p < 0.001); this was restored following supplementation with premenopausal concentrations of oestradiol but not with perimenopausal concentrations of this hormone. Uterus appearances and weights were not altered following administration of Zol (**Supplementary Figure S1**).

**Figure 2:**
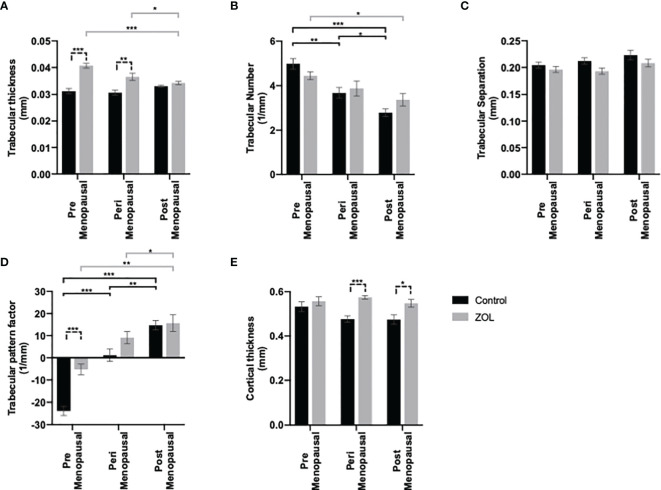
Effects of pre-, post-, and peri-menopausal concentrations of oestradiol ± zoledronic acid on trabecular and cortical bone in mouse tibiae. Twelve-week-old immunocompetent BALB/c mice underwent ovariectomy, and 0, 1.38, or 12.5 mg/L oestradiol was immediately supplemented *via* their drinking water. Three days later, 10 mice from each group received subcutaneous injection of 100 μg/kg zoledronic acid or saline control. All mice received a daily injection of 40 μg/kg goserelin. All data shown are 2 weeks following ovariectomy/oestradiol supplementation. Histograms are all mean ± SD for tibae from 10 mice per group following μCT analysis of trabecular thickness in mm **(A)**, trabecular number **(B)**, trabecular separation **(C)**, trabecular pattern factor **(D)**, and cortical thickness **(E)**. *p < 0.01, **p < 0.001, and ***p < 0.0001.

Oestradiol had significant effects on trabecular bone volume: BV/TV% was higher in the trabecular bone in tibiae of mice following exposure to premenopausal concentrations of oestradiol compared with peri- or post-menopausal concentrations (p < 0.001); BV/TV% was also higher in trabecular bone of tibiae exposed to perimenopausal concentrations of oestradiol compared with pre- or post-menopausal concentrations (p < 0.001 and p < 0.01, respectively) (**Figure 1B**). Administration of Zol caused an increase in trabecular BV/TV% under all concentrations of oestradiol; however, under peri- and post-menopausal concentrations (BV/TV = 13.97% ± 1.13% and 11.45% ± 1.99%, respectively), Zol did not increase bone volume to levels observed under premenopausal oestrogen conditions (BV/TV = 18.70% ± 1.48%; **Figure 1B**). These data suggest that oestradiol has a more potent bone-building effect than Zol in the trabecular area of bone. Interestingly, differences in trabecular bone volume observed in OVX mice following supplementation with pre-, peri-, and post-menopausal concentrations of oestradiol were not due to changes in trabecular thickness (**Figure 2A**). Instead, oestradiol appeared to directly influence trabecular number; mice supplemented with premenopausal concentrations of oestradiol exhibited significantly increased trabecular number compared with mice supplemented with perimenopausal (p < 0.001) or postmenopausal concentrations of oestradiol (p < 0.0001). In contrast, administration of Zol increased trabecular thickness in mice supplemented with pre- (p < 0.0001) and peri- (p < 0.001) menopausal concentrations of oestradiol (**Figure 2A**) but did not alter trabecular number (**Figure 2B**), suggesting that oestradiol and Zol affect bone formation *via* different mechanisms. Trabecular separation was not affected by either oestradiol or Zol (**Figure 2C**). Oestradiol significantly impacted the quality of trabecular bone; trabecular pattern factor was significantly lower under premenopausal concentrations of oestradiol compared with peri- (p < 0.0001) or post- (p < 0.0001) menopausal oestradiol. Interestingly, Zol improved the quality of bone under premenopausal concentrations of oestradiol (p < 0.0001) but had no affect on trabecular pattern factor in trabecular bone under peri- or post-menopausal concentrations of oestradiol (**Figure 2D**).

Effects on cortical bone were less pronounced; cortical thickness was slightly, but not significantly, reduced in mice with peri- and post-menopausal concentrations of oestradiol compared with mice with premenopausal concentrations of oestradiol (cortical bone thickness = 0.047 ± 0.01 mm, 0.47 ± 0.02 mm, and 0.53 ± 0.02 mm, respectively). Furthermore, Zol did not increase cortical bone volume when administered under premenopausal oestradiol but increased cortical volume back to control levels when administered under peri- and post-menopausal oestradiol (cortical bone thickness = 0.55 ± 0.02 mm, 0.57 ± 0.01 mm, and 0.55 ± 0.02 mm, respectively) (**Figure 2E**).

Reduced bone volume observed under postmenopausal concentrations of oestradiol appears to be driven by elevated bone turnover with increased osteoclast number and activity (**Figures 1C, D**) and increased activity of osteoblasts (**Figure 1F**). Alternatively, increased bone volume following administration of Zol was primarily driven by reduced osteoclast activity with only small changes in osteoblast numbers being detected but no changes in activity (**Figures 1C**–**F**). Interestingly, under postmenopausal concentrations of oestradiol, representing conditions in which adjuvant Zol exerts maximum antimetastatic effects in breast cancer patients, administration of Zol reduced the activity of both osteoblasts and osteoclasts (**Figures 1C, D**), implying that Zol may exert increased antiresorptive effects in trabecular bone when oestradiol is low.

### Effects of Oestradiol and Zoledronic Acid on Metastatic Outgrowth of Breast Cancer Cells in Bone

**Figure 3:**
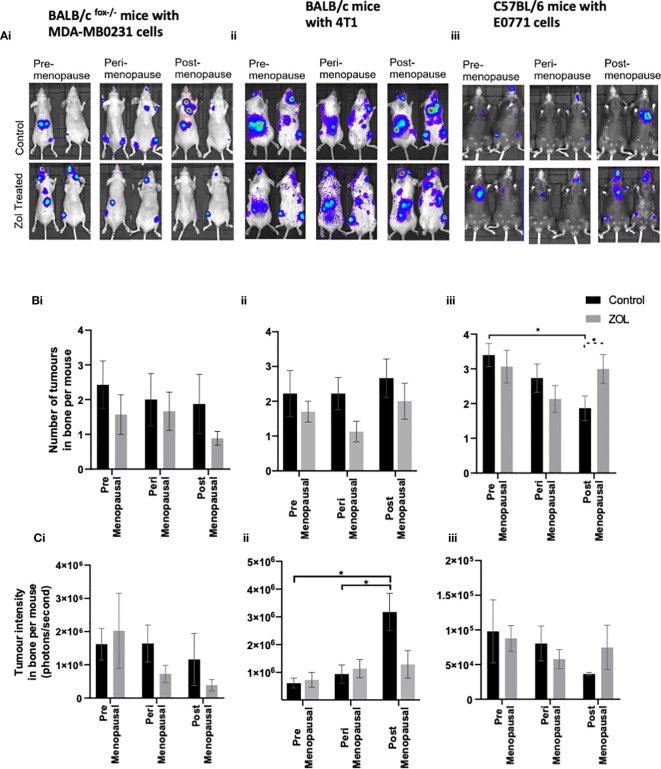
Effects of pre-, post-, and peri-menopausal concentrations of oestradiol ± zoledronic acid on tumour growth in bone. Photographs show representative IVIS images of tumours growing in live animals **(A)**. Histograms show the numbers of overt metastases that form in the skeleton **(B)** and the bioluminescence signal in photons per second (P/S) from Luc2-expressing tumour cells in bone **(C)** in BALB/c fox/- mice injected with MDA-MB-231 cells (i), BALB/c mice injected with 4T1 cells (ii), and C57BL/6 mice injected with E0771 cells (iii) (n = 10/group). Cell lines were disseminated directly into the bloodstream of the mouse *via* intracardiac injection 3, 6, and 7 days after induction of pre-, peri-, or post-menopausal concentrations of oestradiol in BALB/c nude, BALB/c, and C57BL/6, respectively, and 3 days after first injection of zoledronic acid. Zoledronic acid was administered weekly at a dose of 100 μg/kg, and all mice received daily injection of goserelin (40 μg/kg). All data on histograms are represented as mean ± SD; *p < 0.05.

Clinical trials have demonstrated that adjuvant Zol reduced subsequent development of tumour relapse in bone independently of menopausal status or serum oestradiol concentrations (16, 17, 19). We therefore used a variety of *in vivo* models of bone metastasis to see if we could mimic this effect in mice (**Figure 3**). In immunocompromised BALB/c nude mice, oestradiol had no effect on the number of mice developing tumours in bone following i.c. injection of human MDA-MB-231 cells (**Figure 3Bi**) nor did it affect the volume of these tumours (**Figure 3Ci**). Under immunocompromised conditions, there was a trend toward a reduced number of mice developing bone metastasis in Zol-treated mice, and Zol reduced the size of metastatic deposits under peri- and post-menopausal concentrations of oestradiol (**Figures 3Bi, Ci**). Similarly, in immunocompetent BALB/c mice, treatment with Zol resulted in a trend toward a reduced number of bone metastasis following i.c. injection with mouse mammary 4T1 cells; this did not reach significance (**Figure 3Bii**). However, in this model, oestradiol concentration correlated with increased tumour burden, with smaller tumours detected in the bones of mice supplemented with pre- and peri- menopausal concentrations of oestradiol compared with no-oestradiol (p < 0.1). This increase in tumour volume was reduced following administration of Zol (p < 0.1) (**Figure 3Cii**). Interestingly, the opposite effect was observed in immunocompetent C57BL/6 mice injected with mouse mammary E0771 cells. In C57BL/6 mice, circulating concentrations of oestradiol positively correlated with both number and size of bone metastases. Mice supplemented with premenopausal concentrations of oestradiol developed significantly more bone metastases compared with mice not supplanted with oestradiol (p < 0.01; **Figure 3Biii**). In this model, Zol did not reduce bone metastases, instead, under postmenopausal concentrations of oestradiol, mice developed significantly more bone metastases in the Zol-treated compared with the non-Zol-treated groups (p < 0.01; **Figure 3Biii**). These data suggest that Zol has the ability to reduce the outgrowth of metastatic tumour cells in bone, but this is dependent on additional factors including genetic background of the host/tumour.

### Effects of Oestradiol and Zoledronic Acid on Metastatic Outgrowth of Breast Cancer Cells in Soft Tissue

**Figure 4:**
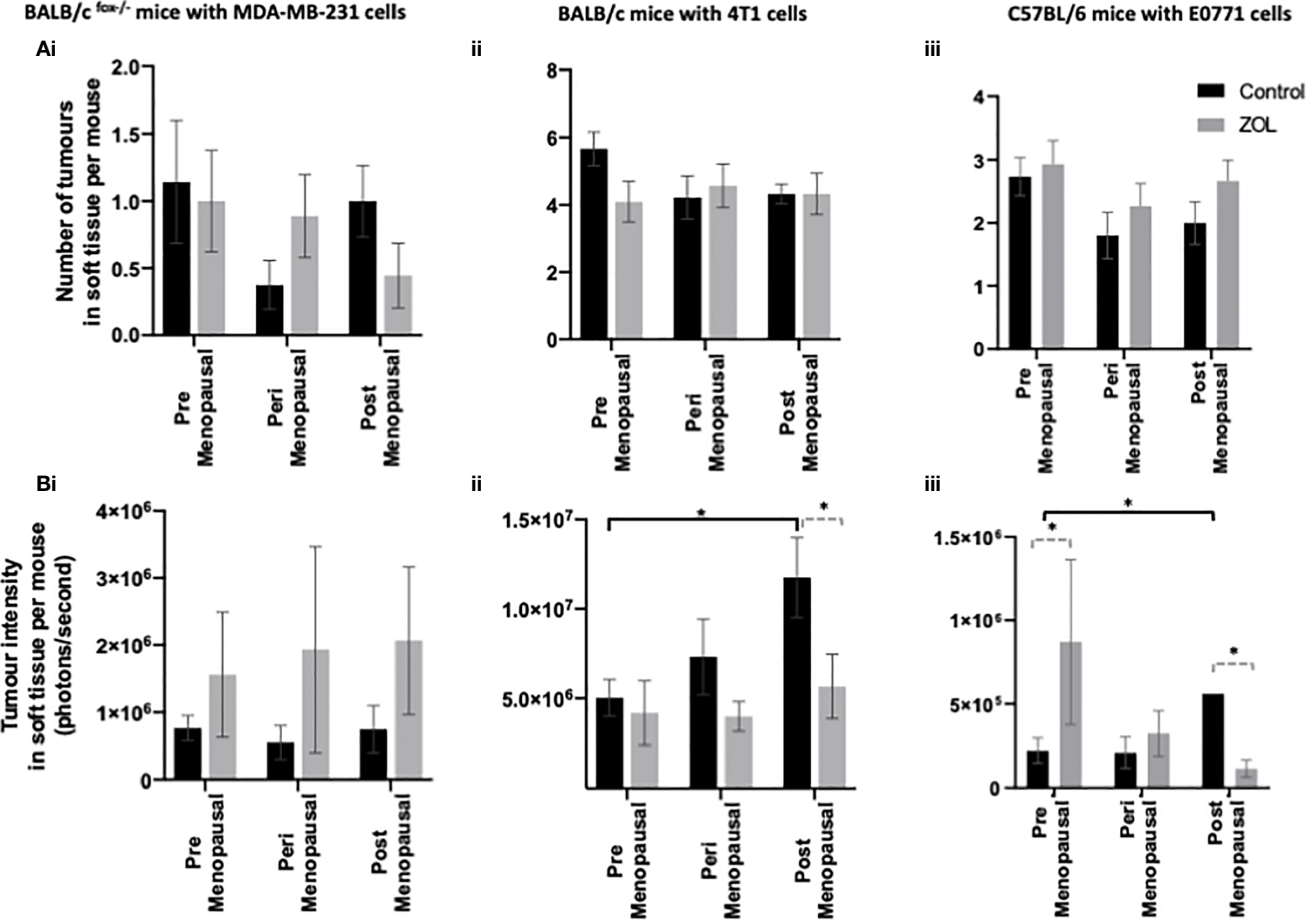
Effects of pre-, post-, and peri-menopausal concentrations of oestradiol ± zoledronic acid on soft tissue metastasis. Histograms show the numbers of overt metastases that form outside the skeleton **(A)** and the bioluminescence signal in photons per second (P/S) from Luc2-expressing tumour cells outside of bone **(B)** in BALB/c fox/- mice injected with MDA-MB-231 cells (i), BALB/c mice injected with 4T1 cells (ii), and C57BL/6 mice injected with E0771 cells (iii) (n = 10/group). Cell lines were disseminated directly into the bloodstream of the mouse *via* intracardiac injection 3, 6, and 7-days after induction of pre-, peri-, or post-menopausal concentrations of oestradiol in BALB/c nude, BALB/c, and C57BL/6, respectively, and 3 days after first injection of zoledronic acid. Zoledronic acid was administered weekly at a dose of 100 μg/kg, and all mice received daily injection of goserelin (40 μg/kg). All data on histograms are represented as mean ± SD; *p < 0.05.

It was hypothesised that under premenopausal conditions, high concentrations of oestradiol may cause tumour cells to move out of the bone microenvironment, driving increased tumour recurrence in soft tissue. Our data show a trend toward reduced numbers of tumours in the soft tissues of mice supplemented with peri- or post-menopausal concentrations of oestradiol compared with mice supplemented with premenopausal concentrations of oestradiol especially in immunocompetent BALB/c and C57BL/6 mice (**Figure 4**). Treatment with Zol had no effect on the number of tumours that developed in mice but did affect tumour volume (**Figure 4B**).

In immunocompromised BALB/c nude mice, oestradiol had no effect on tumour volume, and metastatic tumour growth outside of the skeleton was unaffected by treatment with Zol (**Figure 4Bi**). However, in both immunocompetent BALB/c and C57BL/6 models, oestradiol concentrations inversely correlated with metastatic tumour growth outside of bone. In both of these models, significantly larger tumour volumes were recorded in mice supplemented with postmenopausal concentrations of oestradiol compared with premenopausal concentrations of oestradiol (p < 0.1; **Figures 4Bii, iii**). BALB/c mice treatment with Zol had no effect on tumour growth outside of the skeleton under premenopausal concentrations of oestradiol but significantly reduced tumour volume under postmenopausal concentrations of this hormone (p < 0.01; **Figure 4Bii**). In C57BL/6 mice, treatment with Zol significantly increased tumour volume outside the skeleton (p < 0.05) in mice supplemented with premenopausal concentrations of oestradiol but reduced tumour volume under postmenopausal concentrations of oestradiol (p < 0.05; **Figure 4Biii**). These data suggest that low circulating concentrations of oestradiol stimulate growth of disseminated tumour cells outside of bone and that Zol is only able to exert antitumour effects on extraskeletal metastasis in low oestradiol conditions. Importantly, these effects appear to be dependent on regulation by the adaptive immune system, as neither oestradiol nor Zol affected extraskeletal metastasis in athymic BALB/c nude mice.

**Figure 5:**
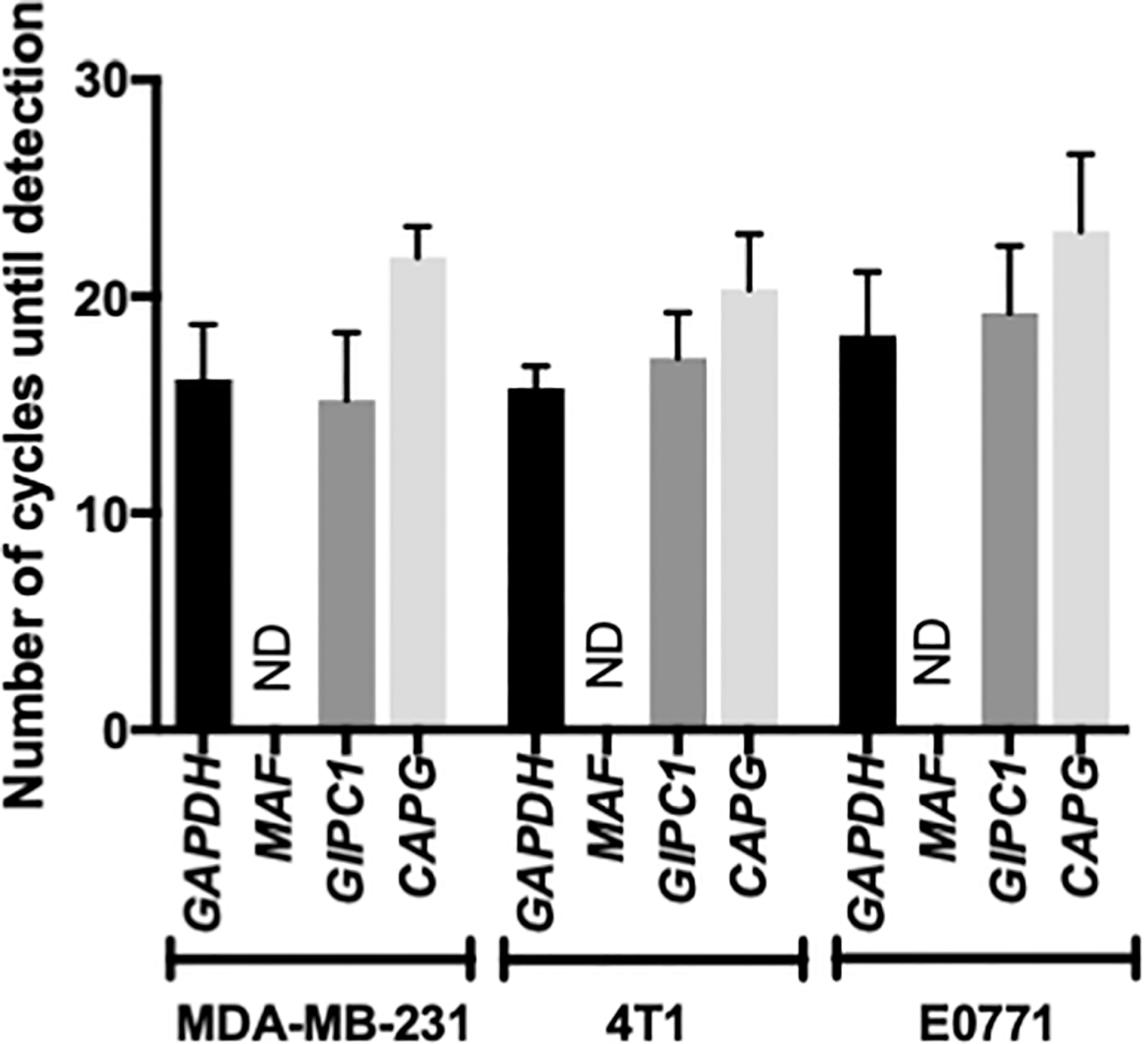
Musculoaponeurotic fibrosarcoma (*MAF*), PDZ domain containing protein GIPC1 (*GIPC1*), and macrophage capping protein (*CAPG*) gene expression profiles in human and mouse bone homing cell lines. Bone homing variants of MDA-MB-231, 4T1, and E0771 cells used in this project were tested for expression of *MAF*, *GIPC1*, and *CAPG*, along with the housekeeping gene glyceraldehyde 3-phosphate dehydrogenase (*GAPDH*) by real-time QPCR. Data shown are mean ± SEM for three repeats of experiments carried out in duplicate. ND, Not Detected.

Previously published studies have indicated that antimetastatic effects of Zol correlate with that tumour cell expression of the transcription factor *MAF* (28), whereas other studies have shown that high expression of *GIPC1* and *CAPG* is associated with antitumour activity of Zol in breast cancer patients (24). We therefore investigated the expression profile of these genes in our breast cancer models. Using Real Time QPCR, *MAF* was not detected within 30 amplification cycles in bone homing, MDA-MB-231, 4T1, or E0771 cells, indicating that this gene is not expressed in these cell lines. In contrast, *CAPG* and *GIPC1* are highly expressed in the bone homing clones used for current experiments, with no significant differences detected (**Figure 5**). *GIPC1* and *CAPG* were detected at 15.22 ± 3.12 and 21.80 ± 1.15 cycles, respectively, compared with 15.22 ± 3.12 for *GAPDH* in MDA-MB-231 cells; in 4T1 cells, GAPC1, *CAPG*, and *GAPDH* were detected at 17.14 ± 2.11, 20.34 ± 2.55, and 16.18 ± 2.33 cycles, respectively; and in E0771 cells, GAPC1 and *CAPG* were detected after 19.225 ± 3.112 and 23 ± 3.562 cycles compared with 18.21 cycles for *GAPDH*.

### Effects of Oestradiol and Zoledronic Acid on the Bone Microenvironment of Tumour-Bearing Mice

**Figure 6:**
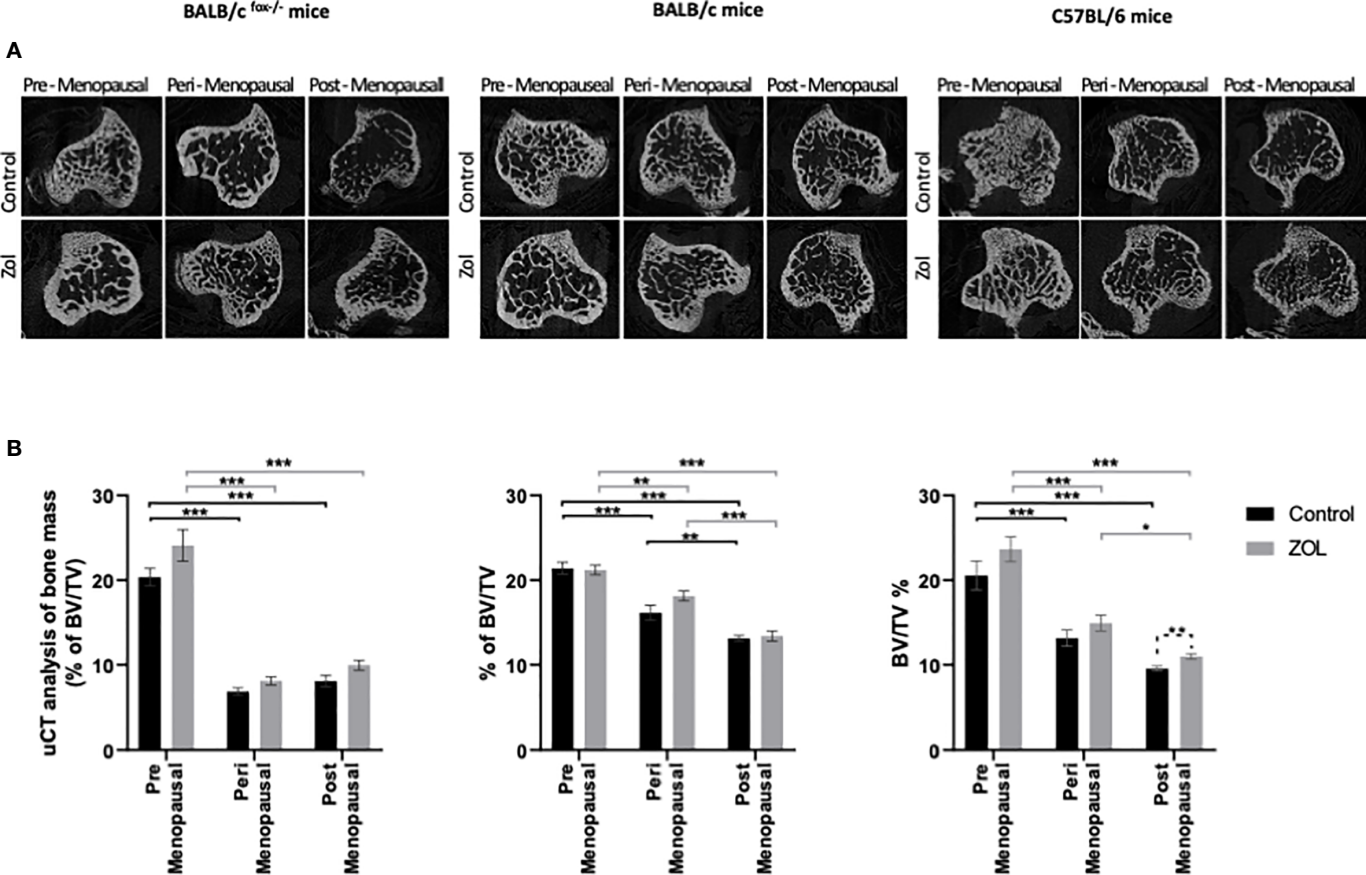
Effects of zoledronic acid on trabecular bone volume in mice supplemented with pre-, post-, and peri-menopausal concentrations of oestradiol following injection of tumour cells. Radiographs show cross-sectional μCT images of trabecular bone **(A)**. Histograms show mean ± SD percentage of trabecular bone volume compared with tissue volume (BV/TV%) **(B)** in BALB/c fox/- mice injected with MDA-MB-231 cells, BALB/c mice injected with 4T1 cells, and C57BL/6 mice injected with E0771 cells (n = 10/group). Cell lines were disseminated directly into the bloodstream of the mouse *via* intracardiac injection 3, 6, and 7 days after induction of pre-, peri-, or post-menopausal concentrations of oestradiol in BALB/c nude, BALB/c, and C57BL/6, respectively, and 3 days after first injection of zoledronic acid. Zoledronic acid was administered weekly at a dose of 100 μg/kg, and all mice received daily injection of goserelin (40 μg/kg). Statistical significance is represented as *p < 0.05, **p < 0.01, and ***p < 0.001.

**Figure 7:**
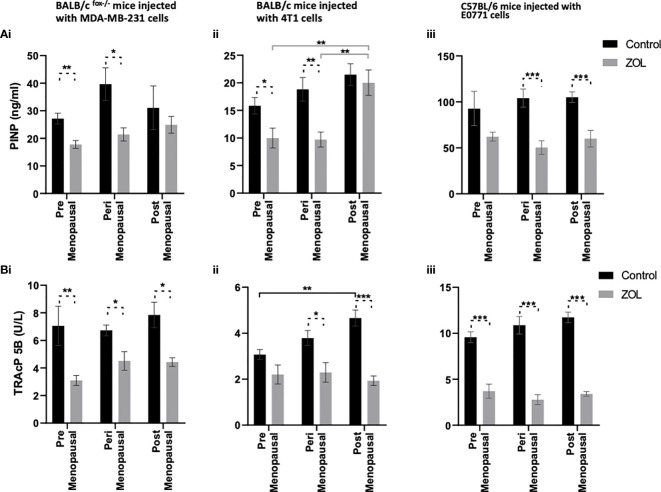
Effects of zoledronic acid on bone cell activity in mice supplemented with pre- peri- and post- menopausal concentration of oestradiol following tumour cell injection. Histograms show osteoblast activity as measured by P1NP ELISA **(A)** and osteoclast activity as measured by TRAcP5 b activity **(B)** from the serum of BALB/c fox/- mice (i), BALB/c (ii) and C57BL/6 (iii) mice injected with MDA-MB-231, 4T1 and E0771 cells respectively (n=10/group). Cell lines were disseminated directly into the blood stream of the mouse via intra-cardiac injection 3, 6 and 7-days after induction of pre- peri or post-menopausal concentrations of oestradiol in BALB/c nude, BALB/c and C57BL/6 respectively and 3 days after first injection of zoledronic acid. Zoledronic acid was administered weekly at a dose of 100ug/kg and all mice received daily injection of goserelin (40ug/kg). All data are shown as mean +/- SD and statistical significance are represented as *P < 0.05, **P < 0.01 and ***P < 0.001.

Evidence from the literature suggests a strong link between bone turnover and metastatic outgrowth in bone (25, 29–31). We therefore investigated whether differential effects of oestradiol and Zol on bone metastasis observed between BALB/c/BALB/c nude and C57BL/6 mice (**Figure 3**) could be explained by effects of this hormone/drug on bone turnover. As previous experiments showed that Zol does not affect oestradiol-induced changes in cortical bone (**Figure 2E**) but has profound effects on oestradiol-induced changes in trabecular bone (**Figures 1**, **2**), which is also the area in which tumours form in mouse models of bone metastasis, we focused our analysis on trabecular bone volume. μCT analysis of trabecular bone from the tibiae of mice revealed that administration of premenopausal concentrations of oestradiol had similar effects on all mouse strains, resulting in BV/TV% of 20.36 ± 1.06 in BALB/c nude, 21.82 ± 0.91 in BALB/c, and 20.22 ± 2.0 in C57BL/6 (**Figure 6**). Reducing oestradiol had significant effects on bone turnover, with the greatest effects observed in BALB/c nude mice in which perimenopausal concentrations of oestradiol reduced trabecular bone volume to 6.38% ± 0.45%, and this remained at a similar volume (8.08% ± 0.70%) under postmenopausal oestradiol. Immunocompetent BALB/c and C57BL/6 exhibited a more gradual response to changes in oestradiol, with significant reductions in bone volume being recorded between mice supplemented with pre- and peri-menopausal concentrations of oestradiol (p < 0.0001 for BALB/c and C57BL/6) and further reductions observed between mice supplemented with peri- and pre-menopausal concentrations of this hormone (p < 0.001 and p < 0.01 for BALB/c and C57BL/6, respectively). Interestingly, in tumour-bearing mice, administration of Zol only increased bone volume in C57BL/6 mice under postmenopausal concentrations of oestradiol (**Figure 6**). In immunocompromised BALB/c nude and immunocompetent BALB/c mice, reduced bone volume observed in mice with peri- and post-menopausal concentrations of oestradiol compared with premenopausal concentrations of this hormone correlated with increased activity of osteoblasts (**Figures 7Ai, ii**) and osteoclasts (**Figures 7Bi, ii**), indicating increased bone turnover. Oestradiol did not appear to alter activity of either osteoclasts or osteoblasts in C57BL/6 mice on termination of this experiment (**Figures 7Aiii, Biii**). Interestingly, administration of Zol reduced osteoblast activity under pre- and peri-menopausal concentrations of oestradiol in BALB/c nude (p < 0.001 and p < 0.01 for pre- and peri-menopausal, respectively) and BALB/c (p < 0.01 and p < 0.001 for pre- and peri-menopausal, respectively) mice. Conversely, in C57BL/6 mice, Zol reduced osteoblast activity under peri- and post-menopausal concentrations of oestradiol (p < 0.001 and p < 0.001, respectively; **Figure 7A**). Zol significantly reduced osteoclast activity in both background strains of immunocompetent and immunocompromised independently of circulating concentrations of oestradiol (**Figure 7B**). These differences observed in the regulation of osteoblast activity may, in part, contribute to the opposing antitumour effects observed in the bones of BALB/c and C57BL/6 mice following administration of Zol (**Figure 3**).

## Discussion

The aim of the current study was to identify if oestrogen was the driver for the differential benefits that adjuvant Zol is having in clinical studies, with postmenopausal breast cancer patients gaining a survival advantage from receiving adjuvant Zol, while premenopausal women do not (16, 17). Multiple clinical trials have demonstrated that pre-, peri-, and post-menopausal women all benefit from reduced bone metastasis when Zol is administered along with adjuvant standard of care. However, in postmenopausal women, this treatment has the added benefit of reducing soft tissue metastasis and increasing survival compared with standard of care alone. The same advantage is not seen in pre- or peri-menopausal women, with some studies showing no reduction in soft tissue metastasis and the AZURE study even suggesting reduced survival following adjuvant Zol in this population (17). Evidence from these clinical trials suggest that the antitumour benefits of adjuvant Zol, outside of bone, are specifically correlated with oestradiol and/or FSH rather than inhibin levels and are independent of tumour subtype (20). We therefore designed the current study to specifically investigate the effects of Zol under pre-, peri-, and post-menopausal concentrations of oestradiol only (FSH low in all) on the outgrowth of metastatic tumour cells, inside and outside of bone. For this study, we selected to use triple-negative human and mouse breast cancer cell lines to negate any direct effects of oestradiol on the cancer cells and to allow us to account for differences in the genetic/immunological background of the host.

We originally hypothesised that administration of Zol under high concentrations of oestradiol may create a bone microenvironment that is unattractive to tumour cells, promoting their relocation from skeletal sites to soft tissue, thus promoting increased soft tissue metastasis observed in the AZURE trial. In order to test this, we built mouse models to mimic serum oestradiol concentrations that are observed in pre-, post-, and peri-menopausal women but with no associated change in serum FSH levels (**Figure 1** and **Supplementary Figure S4**). We found that supplementing OVX mice with 12.5, 1.38, or 0 mg/L resulted in serum concentrations of oestradiol that are comparable to the 40–400, 22–120, and >30 pg/ml found in pre-, peri-, and post-menopausal women, respectively (www.urmc.rochester.edu). Furthermore, adding a daily injection of goserelin prevented production of FSH, thus creating models suitable for studying menopause-specific concentrations of oestradiol. In our models, reducing oestradiol to peri- or post-menopausal concentrations significantly reduced bone volume through increased osteoclast activity, as is also observed in women during the same representative stages of menopause, providing evidence that our model is representative of the human condition (32, 33). Zol has well-characterised properties of inhibiting bone resorption *via* preventing osteoclast activity and causing osteoclast apoptosis in both mouse models and humans (34–36). Our data suggest that the ability of Zol to reduce osteoclastic bone resorption is not affected by oestradiol (**Figures 1**, **7**), and in the absence of tumour, Zol increases bone mass under pre-, peri-, and post-menopausal concentrations of this hormone. Interestingly, despite its anti-osteoclastic properties, administration of Zol to peri- and post-menopausal mice did not restore bone to the same densities observed in premenopausal mice, suggesting that the anabolic effects of oestradiol outweigh the anti-resorptive effects of Zol in our models and that this differential may contribute to the movement of tumour cells from the bone microenvironment to soft tissues in a premenopausal environment. The finding that Zol was only able to elicit anti-resorptive effects in trabecular bone but not cortical bone under premenopausal concentrations of oestradiol is particularly interesting, as the trabecular region is the area of bone that tumour cells home to and grow in mouse models (31, 37).

Reduced bone metastasis observed in breast cancer patients treated with adjuvant Zol was partly reproduced in our mouse models; however, this was dependent on the background strain of the mouse. Mice from a BALB/c background showed a trend toward reduced bone metastasis following administration of Zol, and this was independent of oestradiol concentrations, a functional adaptive immune system, or tumour (human MDA-MB-231 or mouse 4T1). Whereas mice from a C57BL/6 background developed significantly more bone metastases following Zol under postmenopausal concentrations of oestradiol (**Figure 2**). We know that not all breast cancer patients benefit from reduced bone metastasis following adjuvant Zol, and these data indicate that genetic background may be an important factor in determining therapeutic benefit in bone. Evidence from clinical studies has demonstrated that the therapeutic benefit is not determined by oestrogen receptor or human epidermal growth factor receptor 2 (HER2) receptor status of the tumour (16–19). However, separate studies have shown convincing evidence that tumour cell expression of the transcription factor MAF strongly correlates with antimetastatic effects of Zol (28), and combined high expression levels of GIPC1 and CAPG strongly correlate with increased likelihood of developing distant recurrence in bone, with this genotype also conferring increased antitumour sensitivity to adjuvant Zol (24). However, in our mouse models, differences in antimetastatic response to Zol do not appear to be driven by any of these proteins, as *MAF* gene expression below the level of detection by PCR in all three cell lines used and both *CAPG* and *GIPC1* are highly expressed in the bone homing clones used for the current experiments (**Figure 5**), suggesting the importance of other, yet to be identified, genetic determinants in this process. Furthermore, using the MDA-MB-231 model of breast cancer bone metastasis, we and others have previously shown that Zol was only able to elicit an antitumour response in bone under postmenopausal conditions (25, 29, 38). We originally hypothesised that this was due to oestradiol-driven effects on osteoclasts promoting increased bone turnover and stimulating tumour growth, which could be inhibited by reduced activation of osteoclasts (25, 29). This hypothesis initially appeared to be supported by laboratory data showing that reducing osteoclastic bone resorption with the fc fragment of osteoprotegrin (OPGfc) could also inhibit bone metastasis under post- but not pre-menopausal conditions (25). However, increasing clinical evidence suggests that antitumour effects observed in postmenopausal women are only seen following administration of bisphosphonates and not seen following treatment with other osteoclast inhibitors such as denosumab (14). In the aforementioned previously published mouse studies, mice were rendered premenopausal by OVX but not administered goserelin, resulting in both reduced oestradiol and increased FSH. It is therefore possible that FSH and oestrogen may play important roles in the antitumour effects of Zol in bone and that these effects may not primarily be driven by osteoclast-driven alterations to bone resorption. These theories warrant further investigation.

Evidence from preclinical *in vivo* studies has shown that supplementing mice with oestradiol enhanced spontaneous metastasis of ER+ve cells to bone (13), suggesting that high oestradiol may attract tumour cells to bone and may influence whether they survive/become dormant cells in the bone microenvironment or disseminate to other organs. Our current study suggests that, in models of triple-negative breast cancer, premenopausal concentrations of oestradiol are supportive of increased tumour cell dissemination in both bone and soft tissues especially in mice on a C57BL/6 background. Oestradiol did not affect the numbers of tumours that developed in bones or soft tissues of immunocompromised BALB/c nude mice and only partially affected the number of tumour cells that developed in the soft tissues of BALB/c mice. As BALB/c nude mice have no T cells and reduced innate immunity and immunocompetent BALB/c mice elicit less of an immune response when faced with challenge compared with C57BL/6 mice (39), our data suggest that oestrogen effects on antitumour immune response may play important roles in how this hormone regulates tumour cell dissemination and metastatic outgrowth. In agreement with a potential role for the immune response in regulating soft tissue metastases, reducing oestradiol concentrations to those observed in postmenopausal women resulted in significant increases in tumour volume in the soft tissues of immunocompetent BALB/c and C57BL/6 mice but did not affect growth of tumours in the soft tissues of immunocompromised mice (**Figure 3**). Immune response also appeared to be important in the antitumour effects of Zol outside of bone. Zol did not exert antitumour effects in the soft tissues of immunocompromised mice but significantly reduced tumour volume under postmenopausal concentrations of oestradiol in immunocompetent mice (**Figure 3**). Importantly, both immunocompetent models mimicked human clinical trials well, with 4T1 cells in BALB/c mice showing no benefit following administration of Zol and E0771 cells in C57BL/6 mice showing reduced benefit following Zol under premenopausal oestradiol as in the ABCSG-12 and AZURE trials, respectively (16–18). Both oestradiol and Zol have well-documented effects on immune cell regulation. Oestrogen has immunogenic properties, influencing the activity of dendritic cells, macrophages, mast cells, neutrophils, natural killer (NK) cells, B cells, CD4+ T cells, and regulatory T cells (Tregs) (40–42), as well as increasing the protein expression of programmed death ligand 1 (PD-L1) on tumour cells (43). Whereas Zol influences macrophage polarisation in the tumour microenvironment, γδT-cell activation, NK cell activity, Treg activation and infiltration, PD-L1 expression, and T-cell function (44–47). The effects of oestrogen and Zol on the immune system are both profound and often opposing [reviewed in (15)], and it is possible that oestrogen-driven effects on immunomodulation contribute to the differential effects of Zol observed in pre- and post-menopausal women; this hypothesis warrants further investigation.

Taken together, our new data suggest that oestradiol significantly contributes to altered antitumour effects of Zol observed between pre- and post-menopausal women possibly through differing effects on immune cells. However, other genetic and microenvironmental factors including immune regulation are also likely to contribute to the ability of Zol to exert antitumour activity in individual patients.

## Data Availability Statement

The raw data supporting the conclusions of this article will be made available by the authors without undue reservation.

## Ethics Statement

The animal study was reviewed and approved by UK Home Office under licence P99922A2E.

## Author Contributions

PO contributed to the study design, wrote the article, and is the primary grant holder for this project. VC-L and CG performed data acquisition, analysis, preparation of figures, and proofreading of article. CW provided advise on clinical relevance, is a grant holder, and helped edit the article. HC and DL performed data acquisition and analysis. MM provided advise on data analysis and editing of the article. All authors contributed to the article and approved the submitted version.

## Funding

This study was funded by a research grant from Weston Park Cancer Charity (CA168). The IVIS Lumina II system was purchased using an equipment grant from Yorkshire Cancer Research.

## Conflict of Interest

The authors declare that the research was conducted in the absence of any commercial or financial relationships that could be construed as a potential conflict of interest.

## Publisher’s Note

All claims expressed in this article are solely those of the authors and do not necessarily represent those of their affiliated organizations, or those of the publisher, the editors and the reviewers. Any product that may be evaluated in this article, or claim that may be made by its manufacturer, is not guaranteed or endorsed by the publisher.
