# Supplementary figures and images for: Oestradiol Contributes to Differential Antitumour Effects of Adjuvant Zoledronic Acid Observed Between Pre- and Post-Menopausal Women
Source: Front Endocrinol (Lausanne). 2021 Oct 18;12:749428. doi: 10.3389/fendo.2021.749428 (PMC8559775; doi:10.3389/fendo.2021.749428)

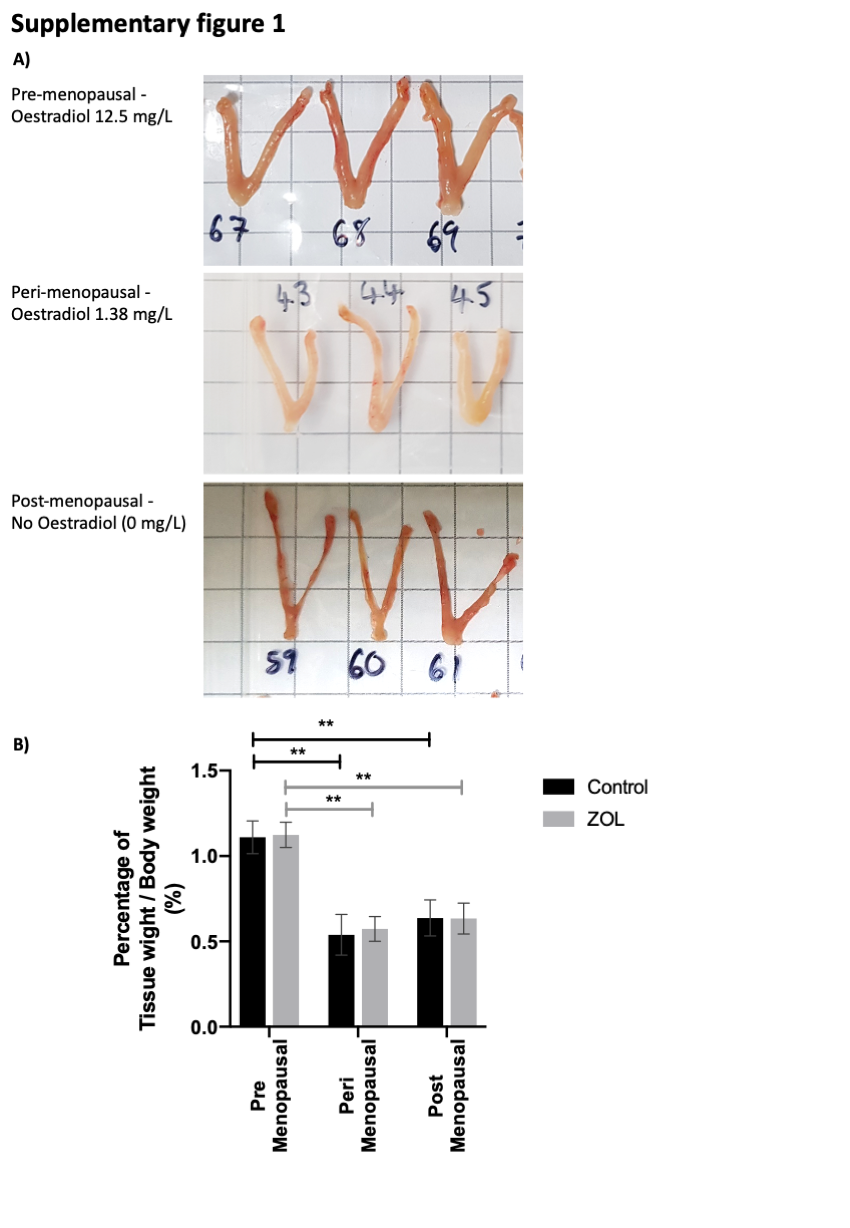

Supplement: Supplementary file 1 [file Image_1.tiff]

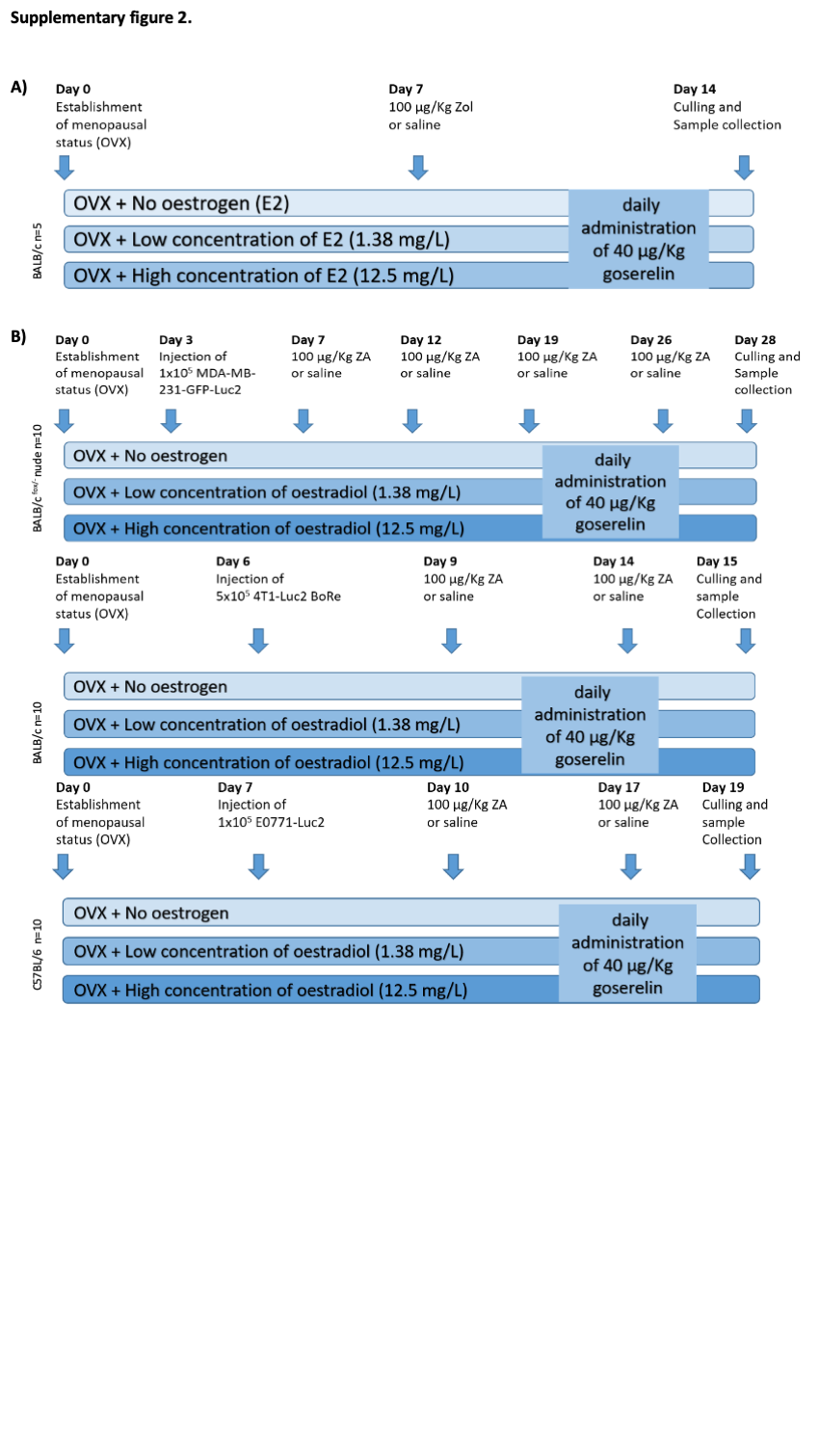

Supplement: Supplementary file 2 [file Image_2.tiff]

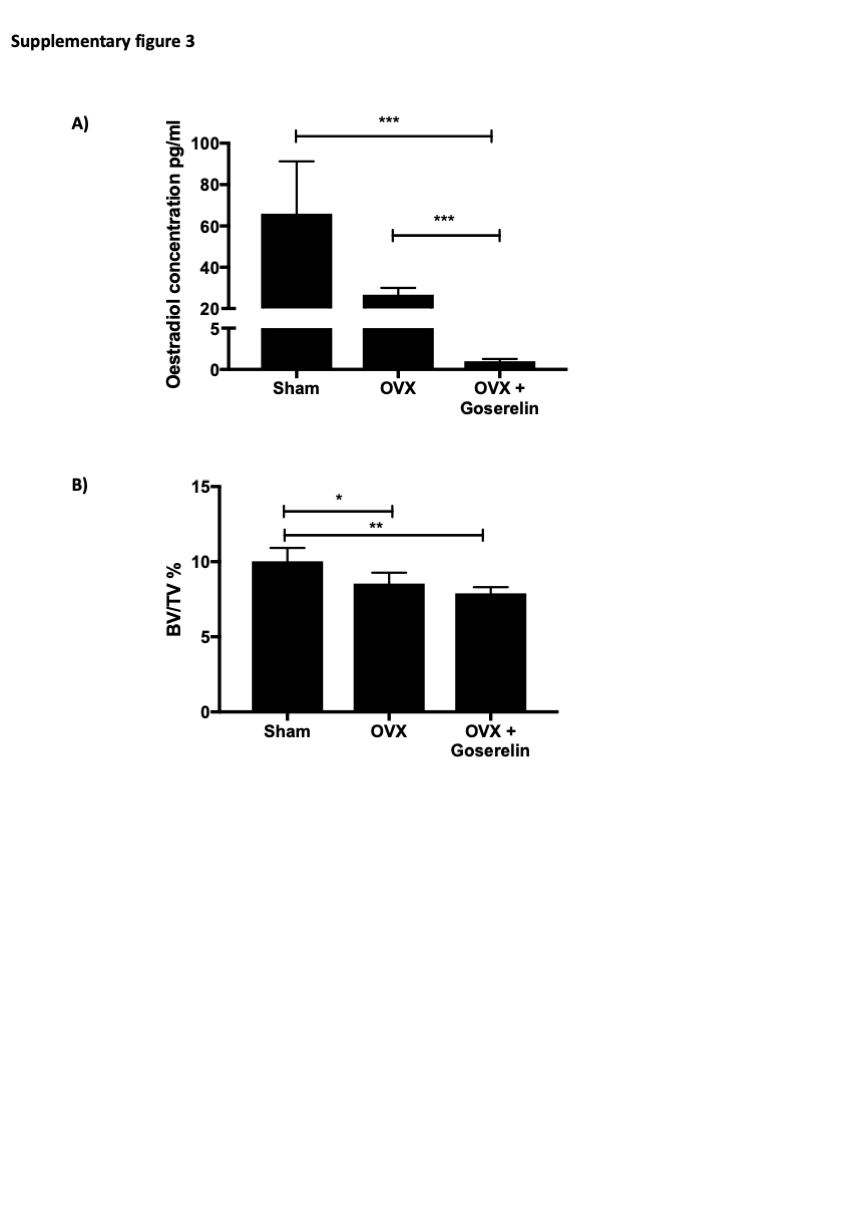

Supplement: Supplementary file 3 [file Image_3.tiff]

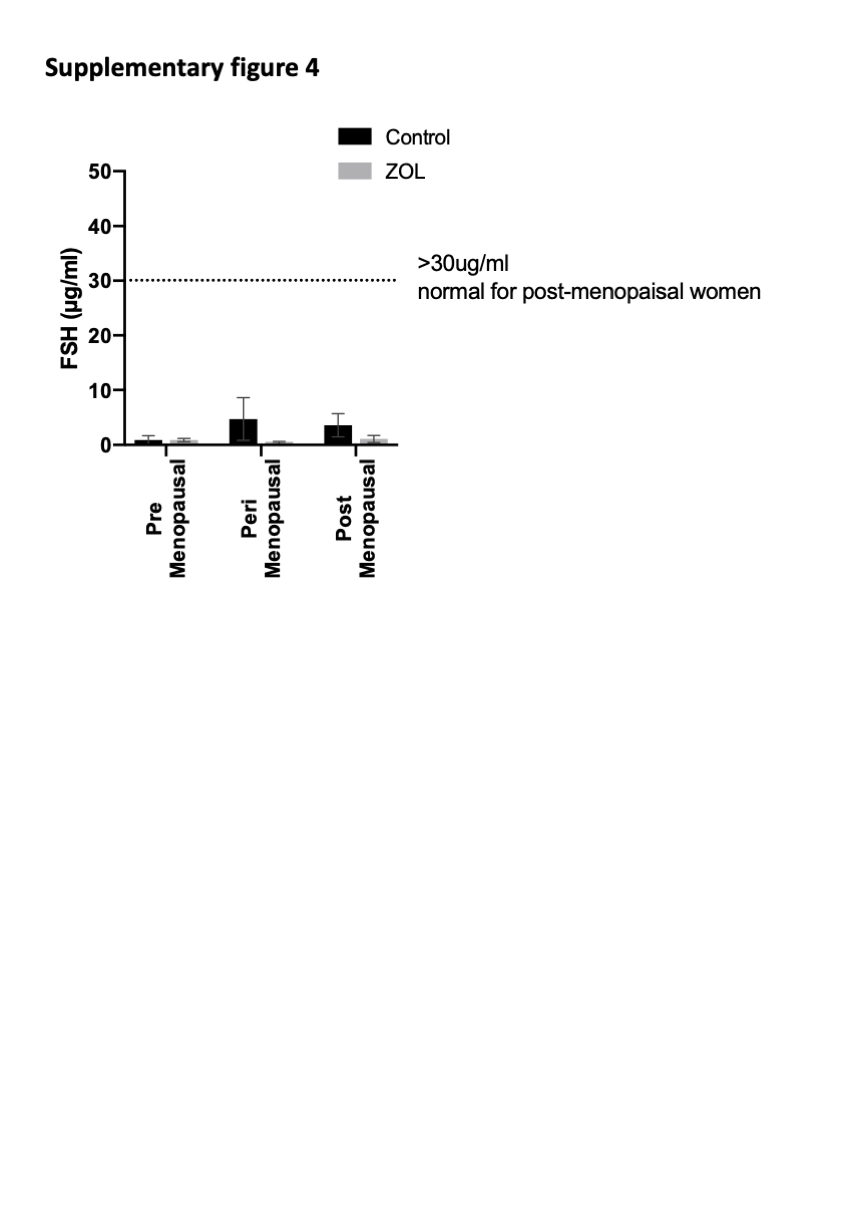

Supplement: Supplementary file 4 [file Image_4.tiff]
